# Genome-wide association study for conformation traits in three Danish pig breeds

**DOI:** 10.1186/s12711-017-0289-2

**Published:** 2017-01-24

**Authors:** Thu H. Le, Ole F. Christensen, Bjarne Nielsen, Goutam Sahana

**Affiliations:** 1 Department of Molecular Biology and Genetics, Center for Quantitative Genetics and Genomics, Aarhus University, Tjele, Denmark; 2 Department of Animal Breeding and Genetics, Swedish University of Agricultural Sciences, Uppsala, Sweden; 3 SEGES Pig Research Centre, Axeltorv, Copenhagen, Denmark

## Abstract

**Background:**

Selection for sound conformation has been widely used as a primary approach to reduce lameness and leg weakness in pigs. Identification of genomic regions that affect conformation traits would help to improve selection accuracy for these lowly to moderately heritable traits. Our objective was to identify genetic factors that underlie leg and back conformation traits in three Danish pig breeds by performing a genome-wide association study followed by meta-analyses.

**Methods:**

Data on four conformation traits (front leg, back, hind leg and overall conformation) for three Danish pig breeds (23,898 Landrace, 24,130 Yorkshire and 16,524 Duroc pigs) were used for association analyses. Estimated effects of single nucleotide polymorphisms (SNPs) from single-trait association analyses were combined in two meta-analyses: (1) a within-breed meta-analysis for multiple traits to examine if there are pleiotropic genetic variants within a breed; and (2) an across-breed meta-analysis for a single trait to examine if the same quantitative trait loci (QTL) segregate across breeds. SNP annotation was implemented through *Sus scrofa* Build 10.2 on Ensembl to search for candidate genes.

**Results:**

Among the 14, 12 and 13 QTL that were detected in the single-trait association analyses for the three breeds, the most significant SNPs explained 2, 2.3 and 11.4% of genetic variance for back quality in Landrace, overall conformation in Yorkshire and back quality in Duroc, respectively. Several candidate genes for these QTL were also identified, i.e. *LRPPRC*, *WRAP73*, *VRTN* and *PPARD* likely control conformation traits through the regulation of bone and muscle development, and *IGF2BP2*, *GH1*, *CCND2* and *MSH2* can have an influence through growth-related processes. Meta-analyses not only confirmed many significant SNPs from single-trait analyses with higher significance levels, but also detected several additional associated SNPs and suggested QTL with possible pleiotropic effects.

**Conclusions:**

Our results imply that conformation traits are complex and may be partly controlled by genes that are involved in bone and skeleton development, muscle and fat metabolism, and growth processes. A reliable list of QTL and candidate genes was provided that can be used in fine-mapping and marker assisted selection to improve conformation traits in pigs.

**Electronic supplementary material:**

The online version of this article (doi:10.1186/s12711-017-0289-2) contains supplementary material, which is available to authorized users.

## Background

Lameness and leg weakness are issues of concern in pig production due to economic and welfare aspects. Leg weakness has been reported as the second most common reason for involuntary culling in pigs for many years, and accounted for 8.6 to 15% of the sows being removed from commercials herds in Nordic countries [1, 2]. Several studies have reported favorable genetic correlations between good legs and litter size and sow stayability [3–5]. Thus, breeding for reduced leg weakness is expected to induce a favorable correlated response on sow reproduction and longevity, and thereby to improve these traits. In fact, conformation traits have been included in breeding goals in almost all Nordic countries, with the aim of reducing lameness [6]. The evaluation of conformation traits has often been carried out subjectively by scoring gait and movement, leg and feet visual observations, and knee and pastern postures [5, 7]. In the literature, heritability estimates for leg conformation traits range from 0.01 to 0.37 [7–10]. These low to moderate heritabilities suggest that faster genetic progress could be achieved by incorporating genetic marker information in the selection process rather than using a traditional pedigree-based selection scheme [11].

Reliability of genomic prediction can be increased by including single nucleotide polymorphisms (SNPs), that are significant in genome-wide association studies (GWAS), in the SNP chips used for routine genomic prediction [12]. Thus, once quantitative trait loci (QTL) associated with conformation traits have been identified, they can be used to improve the reliability of genomic estimated breeding values (GEBV) [13]. In addition, these QTL can be used in fine-mapping studies to identify the causal genetic factors and thus help to understand the biological processes that underlie conformational development in pigs. Information about which genetic factors are involved in the conformation and locomotion of an animal and how much they affect these traits may contribute in setting up a standard scoring system with more objective criteria for uniform evaluation.

Few studies have focused on the mapping of genes for leg conformation traits in pigs. Thus, the objectives of this study were (1) to identify the QTL that are associated with conformation traits in three Danish pig breeds (Landrace, Yorkshire and Duroc) by performing a GWAS; and (2) to examine if the identified genetic variants are associated with multiple conformation traits within one breed and if the same QTL are segregating across breeds by performing meta-analyses. The biological functions of the genes that were closest to the most significant SNP within the detected QTL were also examined to unravel the genetic background of conformation traits.

## Methods

### Animals and scoring of traits

Data on conformation traits from the three Danish breeds, Landrace, Yorkshire and Duroc, which were analyzed in this study, were provided by the Danish pig breeding company DanAvl, Axeltorv, Copenhagen, Denmark (http://www.danavl.dk/). In Denmark, purebred pigs in nucleus herds are performance-tested. Conformation traits are evaluated by trained technicians when the pigs are around five months of age and weigh approximately 100 kg. The data used here were recorded from 2002 to 2015 and included four conformation traits: front leg quality (FRONT), back quality (BACK), hind leg quality (HIND) and overall conformation trait (CONF). The first three traits (FRONT, BACK and HIND) were scored using a three-point scale from 1 to 3, with 3 corresponding to the best conformation. For CONF, a five-point scale from 1 to 5 was used to score the animals, with scores of 1 corresponding to animals that have serious legs or back problems, 3 to average animals, and 5 to animals with excellent conformation. The number of observations in each breed and the means and standard deviation of each trait are in Table 1. Due to the very low frequency of the extreme score categories 1 and 5, the few observations in these categories were merged into the adjacent categories for the association analyses.

**Table 1:** Genotype and phenotype data for the three pig breeds used in this study

Breed	N	8.5 K^a^	60 K^a^	70 K^a^	Trait	Mean (SD)
Landrace	23,989	13,359	10,630	439	FRONT	2.29 (0.45)
					BACK	2.86 (0.35)
					HIND	2.41 (0.49)
					CONF	3.31 (0.66)
Yorkshire	24,130	13,410	10,720	462	FRONT	2.36 (0.48)
					BACK	2.91 (0.27)
					HIND	2.51 (0.50)
					CONF	3.48 (0.60)
Duroc	16,524	7376	9148	270	FRONT	2.32 (0.46)
					BACK	2.79 (0.41)
					HIND	2.32 (0.46)
					CONF	3.09 (0.70)

*N* numbers of animals, *FRONT* front leg quality, *BACK* back quality, *HIND* hind leg quality, *CONF* overall conformation, *SD s*tandard deviation

^a^Number of animals genotyped by different SNP chips: 8.5 K GGP Porcine LD array (GeneSeek^®^), 60 K Illumina PorcineSNP60 BeadChip, 70 K GGP Porcine HD array (GeneSeek^®^)

### Corrected phenotypes

Corrected phenotype, rather than raw phenotype, was used as the dependent variable in the association analysis. Fixed effects used for routine genetic evaluation in DanAvl were included in the following model:

1 $${\mathbf{y}} = {\mathbf{Xb}} + {\mathbf{Zu}} + {\mathbf{e}},$$

where ${\mathbf{y}}$ is a vector of conformation scores, ${\mathbf{b}}$ is a vector of fixed effects including sex, and the combination of herd, year and month at performance testing; body weight at testing performance as covariate; ${\mathbf{u}}$ is a vector of additive genetic values of the animals; ${\mathbf{e}}$ is a vector of the residual effects; ${\mathbf{X}}$ and ${\mathbf{Z}}$ are incidence matrices that associate ${\mathbf{b}}$ and ${\mathbf{u}}$ with ${\mathbf{y}}$. The vectors of random effects ${\mathbf{a}}$ and ${\mathbf{e}}$ were assumed to be normally distributed, i.e. ${\mathbf{u }} \sim N \left( {0,{\upsigma }_{a}^{2} {\mathbf{A}}} \right)$ and ${\mathbf{e}} \sim N\left( {0,{\upsigma }_{e}^{2} {\mathbf{I}}} \right)$ where $\sigma_{a}^{2}$ is additive genetic variance, ${\mathbf{A}}$ is additive relationship matrix derived from pedigree records, $\sigma_{e}^{2}$ is residual variance and ${\mathbf{I}}$ is the identity matrix. The combination of herd, year and month at performance testing also accounts for the effect of the technician who performed the measures, since all records within each level of this combination were recorded by the same person. The analyses were carried out by using the REML method with an R interface to the DMU software package [14]. Heritabilities and genetic correlations between traits were estimated by using a bi-variate linear mixed model based on Model 1. Corrected phenotypes were obtained as the sum of the estimated breeding value and the residual value for each animal from Model 1 and were later used as dependent variables in the association model (Model 2).

### Genotyping and SNP data

Genotyping was carried out using three types of SNP chip: Illumina PorcineSNP60 BeadChip (Illumina, San Diego, CA, USA), and two GeneSeek® custom SNP arrays (Neogen Corporation, Lansing, MI, USA) namely Genomic Profiler (GGP) Porcine LD array featuring over 8500 SNPs (8.5 K) or GGP Porcine HD array, featuring over 70,000 SNPs (70 K). SNPs were quality-controlled within each breed using the following criteria: SNPs with a call-rate lower than 80% across all samples genotyped with each chip or SNPs with a minor allele frequency (MAF) lower than 0.01 were excluded; SNPs that deviated strongly from the Hardy–Weinberg equilibrium (*P* < 10^−7^) and SNPs that were not mapped in the porcine reference genome build Sscrofa10.2 (http://www.ensembl.org/Sus scrofa/Info/Index) were also excluded. Missing genotypes for the remaining SNPs on the 60 K chip were imputed using Beagle version 3.3.2 [15]. After quality control and imputation, 37,080 SNPs, 36,080 SNPs and 32,376 SNPs on the 18 porcine autosomes were retained for the Landrace, Yorkshire and Duroc breeds, respectively. A total of 44,390 different SNPs were available for the three breeds and were later used in the across-breed meta-analysis. The genotype of an animal was removed if the call frequency was less than 90%. The numbers of animals genotyped by different SNP chips and used for the association analysis in each breed are in Table 1.

### Association analyses

#### Single-trait association analysis

 All steps in the single-trait association analyses were carried out using available options in the software Genome-wide complex trait analysis (GCTA) [16].

##### Estimation of the genomic relationship matrix (GRM)

The GRM between individuals within each breed was used to perform principal component analysis and single-trait association analyses. The method that was used to estimate GRM between individuals using SNP data is described in Yang et al. [17]. In this approach, genotype dosages (0, 1, 2) and allele frequency at each SNP were used to calculate the “relationship score” between individual $i$ and $j$ for each SNP. The average “relationship score” across all SNPs was then used as the relationship between individuals $i$ and $j$.

##### Principal component analysis (PCA)

Principal component analysis was performed to assess the population structure within each breed. The top ten eigenvectors with the largest eigenvalues were subsequently included as covariates in the association analysis model to account for the confounding effect of population structure [18]. Including these ten eigenvectors resulted in acceptable genomic inflation factors (lambda-λ) (see Additional file 1: Figure S1, Additional file 2: Figure S2 and Additional file 3: Figure S3). Lambda is one of the standard quality-control measures used for GWAS and reflects the level of inflation of Chi square values when compared with their expectation under the null hypothesis of no association between the trait and SNPs [19].

##### SNP model for single-trait association analysis

Association analyses were carried out using a mixed-linear model with the leaving-one-chromosome-out approach described by Yang et al. [20]. Mixed-linear based association analysis treats the genotype at a single SNP as a fixed effect and the additive polygenic effect as a random effect, and will be called the SNP model (Model 2):

2 $${\mathbf{y}}_{c} = \mathbf{1}\mu + {\mathbf{Xb}} + {\mathbf{S}}\alpha + {\mathbf{Zu}} + {\mathbf{e}},$$

where ${\mathbf{y}}_{c}$ is a vector of corrected phenotypes obtained from Model 1; **1** is a vector of 1s; $\mu$ is the general mean; ${\mathbf{X}}$ is a matrix containing the 10 eigenvectors that were derived from PCA and included as covariates, and ${\mathbf{b}}$ is a vector of associated effects; ${\mathbf{S}}$ is the design matrix that contains the allele contents at the fitted SNP, i.e. counts (0, 1, 2) of the second allele, and $\alpha$ is the allele substitution effect; ${\mathbf{u}}$ is a vector of additive polygenic effect, and ${\mathbf{Z}}$ is an incidence matrix associating ${\mathbf{u}}$ with ${\mathbf{y}}$. Vectors of random effects ${\mathbf{u}}$ and ${\mathbf{e}}$ were assumed to be normally distributed, i.e. ${\mathbf{u}} \sim N\left( {0,\sigma_{g}^{2} {\mathbf{G}}} \right)$ and ${\mathbf{e}} \sim N\left( {0,\sigma_{e}^{2} {\mathbf{I}}} \right)$, where $\sigma_{g}^{2}$ is polygenic variance, ${\mathbf{G}}$ is the GRM, $\sigma_{e}^{2}$ is residual variance and ${\mathbf{I}}$ is the identity matrix. In the “leaving-one-chromosome-out” approach association analysis is performed for a candidate SNP using a ${\mathbf{G}}$ that is computed without including the chromosome on which the candidate SNP is located. For instance, an association analysis between a SNP on chromosome 1 used a ${\mathbf{G}}$ that was computed only from SNPs on chromosomes 2 to 18. This approach avoids “double-fitting” of the candidate SNP into the model (both as a fixed effect and a random effect) which can reduce power, as demonstrated by Listgarten et al. [21].

##### Multiple testing correction

The Bonferroni correction was applied to correct for multiple testing. Since the number of SNPs was relatively similar for the three breeds, the Bonferroni correction threshold was calculated for all three breeds based on the number of SNPs for the Landrace breed, i.e. 0.05/36,080 = 1.38 × 10^−6^. Thus, the 5% genome-wide significance level used to avoid type I errors was 1.38 × 10^−6^ for all three breeds.

#### Meta-analyses

##### Within-breed multi-trait meta-analysis

A multiple trait meta-analysis was performed within each breed using the approximate multi-trait test statistic described by Bolormaa et al. [22]. Effects of a SNP across all traits were calculated and combined with the genomic correlation matrix between traits to perform a multi-trait Chi square test with the number of degrees of freedom equal to the number of traits. The formula to calculate the multi-trait statistic for each SNP was as follows:

$$Multi\text{-}trait \chi^{2} = {\mathbf{t}}_{{\mathbf{i}}}^{\prime } {\mathbf{V}}^{ - 1} {\mathbf{t}}_{{\mathbf{i}}} ,$$

where ${\mathbf{t}}_{{\mathbf{i}}}$ is a vector of signed t-value of SNP_i_ across traits (t-value = SNP effect/standard error of SNP effect), ${\mathbf{V}}^{ - 1}$ is the inverse matrix of the genomic correlation matrix between traits calculated from these t-values. The significance threshold from the single-trait association analyses (i.e. *P* value <1.38 × 10^−6^) was applied for these within-breed multi-trait analyses.

##### Across-breed meta-analysis

Meta-analyses across the three breeds for a trait was carried out using the METAL software developed by Willer et al. [23]. The direction of the effect and the *P* values from each study *i* were converted into z-scores. These z-scores for each SNP were weighted by the sample size of each study and combined in a weighted sum across breeds. The test statistic follows the standard normal distribution and was calculated as follows:

$$Z = \frac{{\mathop \sum \nolimits_{i}\Phi ^{ - 1} \left( {\frac{{P_{i} }}{2}} \right)*sign\left( {\Delta _{i} } \right)*\sqrt {N_{i} } }}{{\sqrt {\mathop \sum \nolimits_{i} N_{i} } }},$$

where $P_{i}$ is the *P* value for study *i*; $\Delta _{i}$ is the direction of effect for study *i*; and $N_{i}$ is the sample size for study *i*. A Bonferroni correction was applied with a significance threshold of 0.05/44,390 = 1.13 × 10^−6^ for all four traits.

### QTL characterization

#### QTL region

Since the software used does not report model convergence, SNPs with an allele substitution effect estimate that fell outside the range of ±2SD of the corrected phenotypes were filtered out to avoid their probably unrealistic large effect due to inappropriate convergence in the parameter estimation. Also, only SNPs that passed the Bonferroni correction threshold were selected to mark the QTL region. A QTL region was defined by extending the position of the most significant SNP (top SNP) on either side until all SNPs within that region had a $- log_{10} \left( {P\text{-}value} \right)$ higher than the $- log_{10} \left( {P\text{-}value} \right)$ of the top SNP minus 3 units.

#### Size of the QTL effect

The size of the effect of a QTL, which was defined as the contribution of the most significant SNP within that QTL to the phenotypic variance of the trait, was calculated as $\left( {2pq\beta^{2} /\sigma_{P}^{2} } \right)$, in which $p$ and $q$ are the allele frequencies, $\beta$ is the estimated SNP effect, and $\sigma_{P}^{2}$ is the phenotypic variance of the trait. The corresponding contribution to the genetic variance for that SNP was calculated as a proportion of the genetic variance $\sigma_{a}^{2}$.

### Candidate genes underlying the QTL

Genes that harbored the most significant SNPs within each QTL region from single-trait analyses and across-breeds meta-analyses were searched based on the pig genome assembly, Sscrofa10.2 (http://www.ensembl.org/Susscrofa/Info/Index).

## Results

### Descriptive statistics

Means and standard deviations for each trait in the Landrace, Yorkshire and Duroc breeds are in Table 1 (see “Methods” section). Means for CONF were slightly above the middle score 3 and ranged from 3.09 to 3.31, with the standard deviations ranged from 0.60 to 0.70 across the three breeds. Means for FRONT, BACK and HIND were all significantly greater than the middle score 2. The trait BACK had the highest means (from 2.79 to 2.91) and low standard deviations (from 0.27 to 0.41) which indicate that most animals obtained the highest score i.e. 3 for this trait. For FRONT and HIND, means ranged from 2.29 to 2.36 and from 2.32 to 2.51, with standard deviations ranging from 0.45 to 0.48 and from 0.46 to 0.50, respectively.

Heritability estimates in each breed are presented on the diagonals in Table 2. In general, estimated heritabilities were low for all traits, ranging from 0.02 to 0.13. The trait CONF had a slightly higher heritability than the other traits in all three breeds.

**Table 2:** Heritabilities and correlations between traits in Landrace, Yorkshire and Duroc

Breed	Trait	FRONT	BACK	HIND	CONF
Landrace	FRONT	*0.02* (*0.00*)	0.10	0.34	0.54
	BACK	0.48 (0.15)	*0.09* (*0.01*)	0.14	0.49
	HIND	0.75 (0.15)	0.23 (0.10)	*0.06* (*0.01*)	0.79
	CONF	0.87 (0.09)	0.60 (0.06)	0.96 (0.03)	*0.12* (*0.01*)
Yorkshire	FRONT	*0.03* (*0.01*)	0.12	0.30	0.54
	BACK	0.46 (0.14)	*0.09* (*0.01*)	0.20	0.45
	HIND	0.72 (0.13)	0.52 (0.10)	*0.06* (*0.01*)	0.82
	CONF	0.76 (0.09)	0.66 (0.07)	0.97 (0.02)	*0.10* (*0.01*)
Duroc	FRONT	*0.05* (*0.01*)	0.23	0.44	0.62
	BACK	0.55 (0.13)	*0.08* (*0.01*)	0.21	0.59
	HIND	0.95 (0.09)	0.55 (0.12)	*0.07* (*0.01*)	0.73
	CONF	0.95 (0.05)	0.73 (0.07)	0.94 (0.03)	*0.13* (*0.02*)

Within the breed: heritabilities (on diagonal, in italic), genetic correlations estimated from bi-variate linear mixed model (lower diagonal) and genomic correlations estimated from signed t-values of all SNPs between traits (upper diagonal) which was matrix **V** in within-breed multi-trait meta-analysis

Standard errors are in parenthesis

*FRONT* front leg quality, *BACK* back quality, *HIND* hind leg quality, *CONF* overall conformation

Genetic correlations were estimated using the linear mixed model (Model 1) and genomic correlations were estimated from signed t-values of all SNPs between traits in each breed; they are presented on the lower and upper diagonals, respectively in Table 2. The traits FRONT, HIND and CONF were highly correlated with the genetic correlations ranging from 0.72 to 0.97. The trait BACK seems to be genetically more different from the other three traits, especially in the Landrace and Yorkshire breeds, for which the estimated genetic correlations ranged from 0.23 to 0.66. When breeds were compared, genetic correlations between traits were higher in Duroc than in Landrace and Yorkshire. Regardless of the breed, genomic correlations between traits followed the same pattern as the genetic correlations but at lower magnitudes. For instance, genomic correlations of BACK with the other traits ranged from 0.14 to 0.59.

### Single-trait association analyses

Genomic regions that were found to be associated with conformation traits for Landrace, Yorkshire and Duroc are in Table 3, together with the candidate genes and the top associated SNPs within each region. Visual overviews of the location of the QTL are in the Manhattan plots of Additional file 1: Figure S1, Additional file 2: Figure S2 and Additional file 3: Figure S3. QTL regions were identified on *Sus scrofa* chromosome (SSC) 1, 2, 3, 4, 5, 6, 7, 10, 12, 13 and 18. In general, the number of associated regions was larger for CONF than for the other traits.

**Table 3:** QTL regions for conformation traits and the most significant SNP within each region in three pig breeds

Trait	n	Chr	QTL region		Most significant SNP			Candidate genes^b^
			Right position (bp)	Left position (bp)	SNP^a^	*P* value	Effect size (%)	
*Landrace*								
BACK	41	1	63,345,571	68,207,754	rs80850790	3.53 × 10^−8^	0.12	SRSF12, PNRC1
		1	81,835,391	105,362,894	rs81287678	1.48 × 10^−8^	0.13	*SLC14A2*
		3	117,911,196	118,765,898	rs81258584	1.64 × 10^−7^	0.11	*PPM1G*
		5	67,981,416	68,326,348	rs80985094	9.93 × 10^−8^	0.14	*CCND2*
		7	36,202,231	37,157,566	rs80828473	7.41 × 10^−12^	0.19	*PPARD*
HIND	25	1	216,980,027	242,984,908	rs80946156	5.21 × 10^−8^	0.13	*RCL1*
CONF	104	3	97,726,517	97,741,803	rs81303888	2.72 × 10^−7^	0.11	*LRPPRC*
		3	117,911,196	117,911,196		2.42 × 10^−7^	0.11	BRE, ENSSSCG00000026465
		5	67,518,456	69,897,370	rs81384722	2.29 × 10^−8^	0.15	*ENSSSCT00000000782*
		6	74,354,607	84,927,021	rs81389032	1.84 × 10^−10^	0.17	ENSSSCT00000032147, ENSSSCT00000003913
		7	21,053,530	37,812,119	rs80828473	6.28 × 10^−11^	0.18	*PPARD*
		7	131,126,819	131,126,819	rs81397155	6.20 × 10^−8^	0.15	TDRD9, CEP170B
		12	24,812,751	26,022,727	rs81312521	3.16 × 10^−8^	0.13	*NGFR*
		12	46,857,615	47,352,945	rs81435770	2.09 × 10^−7^	0.11	*PIPOX*
*Yorkshire*								
FRONT	2	7	52,843,780	52,860,434	rs80995679	1.27 × 10^−6^	0.09	CHRNB4, PSMA4
BACK	2	6	142,725,268	142,803,333	rs81305243	1.08 × 10^−6^	0.10	DAB1
HIND	57	1	92,766,049	101,369,745		5.66 × 10^−8^	0.12	RIPPLY2, SNAP91
		1	198,725,106	205,843,789	rs80999532	1.41 × 10^−9^	0.15	TMX1, FRMD6
		7	103,101,452	121,513,304	rs80894106	3.36 × 10^−8^	0.12	VRTN, SYNDIG1L
CONF	222	1	92,766,049	92,766,049		4.99 × 10^−12^	0.20	RIPPLY2, SNAP91
		1	198,725,106	208,707,283	rs80783847	2.42 × 10^−13^	0.22	ENSSSCT00000005518
		6	41,235,400	43,110,092	rs81395827	3.82 × 10^−9^	0.15	ZNF382
		7	102,881,143	103,495,170	rs80894106	3.86 × 10^−11^	0.18	VRTN, SYNDIG1L
		10	18,665,600	18,920,852	rs81309142	8.35 × 10^−8^	0.12	ENSSCG00000030502, ZBTB18
		12	14,208,800	15,365,718	rs81440562	5.63 × 10^−12^	0.20	CD79B, GH1
		18	10,626,879	14,885,152	rs81469271	4.39 × 10^−9^	0.15	*ENSSCG000000288580*
*Duroc*								
FRONT	12	3	97,469,056	99,288,792	rs323557679	3.28 × 10^−7^	0.16	*MSH2*
		6	47,212,157	57,685,509	rs81226413	4.22 × 10^−8^	0.18	*ENSSCT00000003685*
BACK	128	3	100,232,086	100,448,894	rs81373717	6.41 × 10^−31^	0.85	EPAS1, PRKCE
		6	47,212,157	53,526,888	rs338147539	2.59 × 10^−8^	0.19	*CCDC61*
HIND	31	6	54,949,995	60,446,765	rs81327648	9.17 × 10^−11^	0.25	*WRAP73*
CONF	235	2	36,580,946	46,128,234		6.01 × 10^−8^	0.16	ANO3
		3	97,469,056	100,477,666	rs81373756	6.44 × 10^−20^	0.54	*PRKCE*
		4	99,503,615	111,556,304	rs81382406	3.88 × 10^−8^	0.17	*CHRNB2*
		6	47,573,330	57,685,509	rs81333163	5.30 × 10^−20^	0.52	A1BG, RPS5
		7	34,755,602	36,697,937	rs80852624	9.4 × 10^−8^	0.18	HMGA1, RPS10
		7	103,495,170	103,495,170	rs80894106	8.60 × 10^−11^	0.25	VRTN, SYNDIG1L
		12	25,298,982	25,580,071	rs327303574	3.80 × 10^−11^	0.26	ENSSCT00000022825, B4GALNT2
		13	132,640,404	133,032,413		1.21 × 10^−6^	0.14	*IGF2BP2*

*FRONT* front leg quality, *BACK* back quality, *HIND* hind leg quality, *CONF* overall conformation, *n* number of significant SNPs, Chr *Sus scrofa* chromosome, *Effect size* percentage of phenotypic variance explained by the most significant SNP

^a^ *SNP* rsID

^b^ *Candidate genes* in which the most significant SNPs located are in italic

In total, 14 regions were significantly associated with conformation traits in Landrace, of which five were associated with BACK, one with HIND, and eight with CONF, but none with FRONT (Table 3). The most significant SNP identified for the Landrace breed, rs80828473, was located at 36.2 Mb within the *PPARD* gene on SCC7. This SNP explained 0.2% of phenotypic variance (2% of genetic variance) of BACK. The second most significant SNP, rs81389032 (SSC6: 74.4 Mb), was not within any reported coding gene, but was located in a region between the genes *ENSSCT00000032147* and *ENSSCT00000003913*.

For the Yorkshire breed, 12 regions were associated with the traits analyzed: one region with FRONT, one with BACK, three with HIND, and seven with CONF (Table 3). The most significant SNP was rs80783847 (SSC1: 199.4 Mb) and was located close to gene *ENSSSCT00000005518*; explained 0.2% of the phenotypic variance (2.3% of the genetic variance) of CONF. The next two most significant SNPs were located between the genes *RIPPLY2* and *SNAP91* (SNP on SSC1: 92.7 Mb), and between the genes *CD79B* and *GH1* (SNP rs81440562 on SSC12: 15.0 Mb).

Among the 13 QTL regions identified for the Duroc breed, the region on SSC3 between 100.2 and 100.4 Mb showed the highest peak (Table 3). The most significant SNP in this region, rs81373717, contributed 0.9% of the phenotypic variance (11.4% of the genetic variance) of BACK. This SNP was not located within any coding gene but was between the genes *EPAS1* and *PRKCE*. The next two most significant SNPs were rs81373756 (SSC3: 100.4 Mb) within *PRKCE* and rs81333163 (SSC6: 57.6 Mb) between *A1BG* and *RPS5*. They were both associated with CONF and each explained 0.5% of the phenotypic variance of this trait.

Some regions and genes showed significant association with more than one trait within a breed, suggesting the presence of a variant that may affect multiple conformation traits. For example, strong associations were found: (1) *PPARD* (SSC7: 36.2 Mb) was highly associated with both BACK and CONF in Landrace; (2) the QTL region located between *RIPPLY2* (92.6 Mb) and *SNAP91* (92.8 Mb) on SSC1 exhibited high association with both CONF and HIND in Yorkshire; and (3) the region that comprises *PRKCE* (SSC3: 100.5 Mb) showed high association with both CONF and BACK in Duroc. In the single-trait within-breed association analyses, the only region that overlapped between breeds was on SSC7 and it was associated with CONF in Yorkshire and Duroc.

### Meta-analyses

#### Within-breed multi-trait meta-analysis

**Fig. 1:**
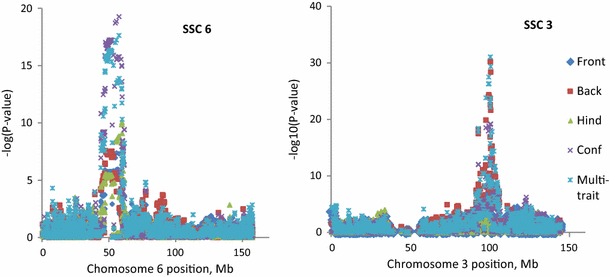
The $- \varvec{log}\left( {\varvec{P}\text{-}\varvec{value}} \right)$ of SNPs on SSC6 and SSC3 from single-trait and multi-trait meta-analyses in Duroc

The numbers of significant SNPs identified in within-breed multi-trait meta-analyses for Landrace, Yorkshire and Duroc (206, 257 and 306 SNPs, respectively) were larger than in single-trait analyses (not shown). Their distribution on the genome was showed in the Manhattan plots in Additional file 4: Figure S4. Many of the SNPs that were found significantly associated with more than one trait in the single-trait analyses were confirmed in the multi-trait analyses, which suggests that the QTL containing these SNPs have pleiotropic effects. For instance, Fig. 1 shows the significance of the effects of SNPs on SSC6 and SSC3 from single-trait and multi-trait analyses in Duroc, which suggests that the corresponding QTL regions on these chromosomes affect multiple traits. Multi-trait meta-analysis can increase the power of QTL detection, since in general SNPs had lower P-values in the multi-trait analyses than in the single-trait analyses. Table 4 presents examples of such lower P-values for the most significant SNPs detected in the multi-trait analyses compared with the single-trait analyses.

**Table 4:** Most significant SNPs detected in the within-breed multi-trait meta-analyses in three pig breeds

Breed	Chr	SNP^a^	Position bp	*P* value	Trait	Single-trait *P* value^b^
Landrace	6	rs81389032	74,418,977	4.08 × 10^−17^	CONF	1.84 × 10^−10^
Yorkshire	1	rs80783847	199,414,449	3.48 × 10^−14^	HIND	1.43 × 10^−9^
	1				CONF	2.42 × 10^−13^
Duroc	3	rs81373717	100,232,086	9.26 × 10^−32^	BACK	6.42 × 10^−31^
	3				CONF	7.97 × 10^−20^

*Chr Sus scrofa* chromosome, *FRONT* front leg quality, *BACK* back quality, *HIND* hind leg quality, *CONF* overall conformation

^a^ *SNP* rsID

^b^ *P* value from single-trait association analysis

#### Meta-analysis across breeds by trait

**Fig. 2:**
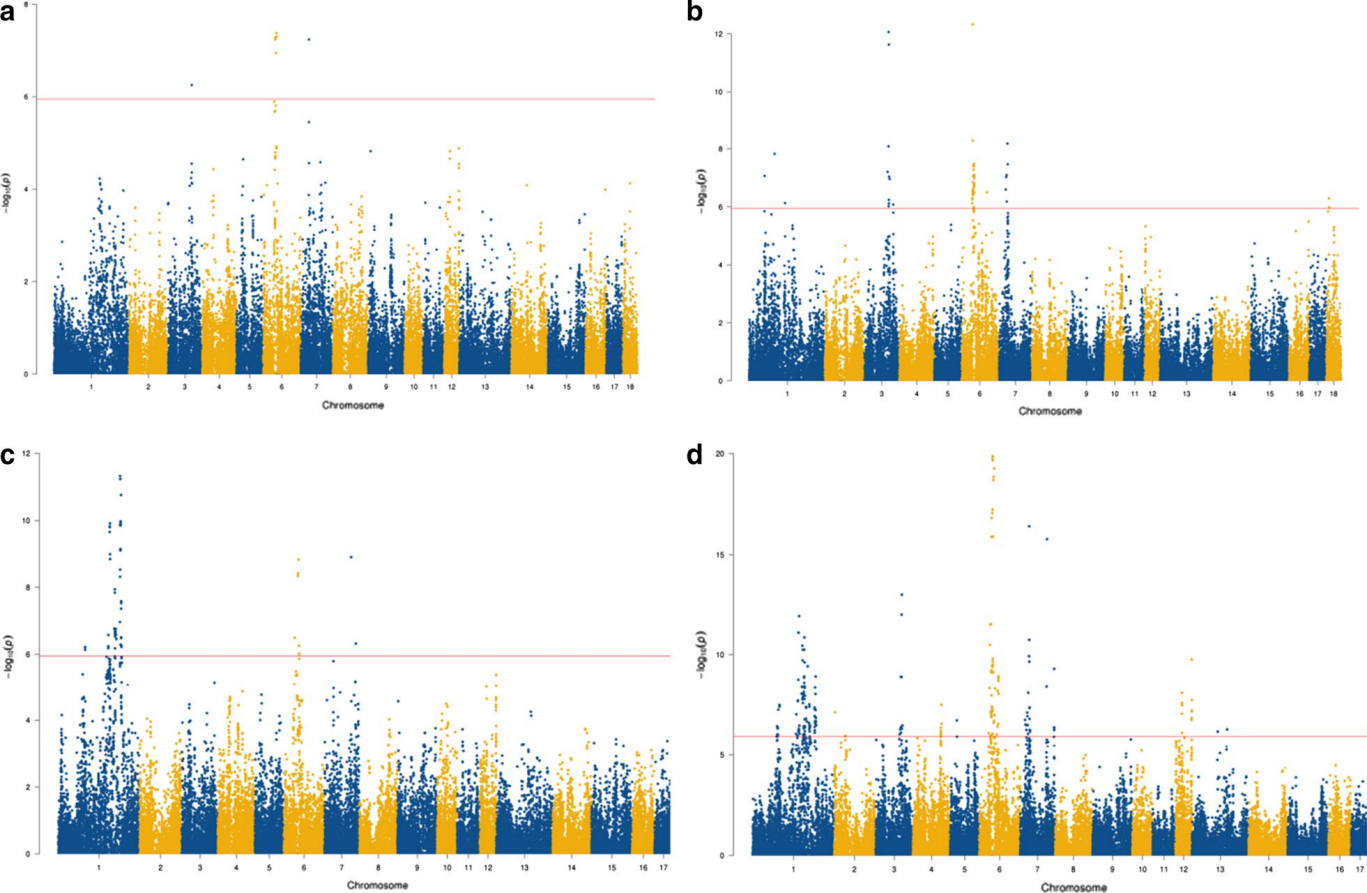
Manhattan plots of across-breed meta-analyses for **a** FRONT, **b** BACK, **c** HIND and **d** CONF. x-axis represents the chromosomes and y-axis represents ${-}log_{10} \left( {P\text{-}value} \right)$. The *red line* indicates genome-wide significance threshold at *P* value <1.13 × 10^−6^

In total 36 regions were associated with the traits analyzed in the across-breed meta-analyses: three regions were associated with FRONT, eight with BACK, seven with HIND and 18 with CONF [Fig. 2 and Additional file 5: Table S1]. Among these 36 regions, several QTL regions that were detected in the single-trait within-breed analysis were confirmed and several additional regions with novel candidate genes were identified. For instance, the most significant SNP associated with CONF, rs81344309 (SSC6: 52.0 Mb), which is located between the two coding genes *ENSSCG00000003243* and *ZNF614*, was not identified in the single-trait analyses. Similarly, the two second most significant SNPs are also new and are intergenic on SSC7: rs342640079 (34.9 Mb) between *GRM4* and *HMGA1* and rs80894106 (103.5 Mb) between *VRTN* and *SYNDIG1L*. The latter SNP is associated with CONF in both Yorkshire and Duroc in the single-trait analyses, but reached the higher level of significance in the across-breed meta-analysis.

## Discussion

### Single-trait association analysis

The present GWAS detected several chromosomal regions that are associated with conformation traits in the three pig breeds, and in many cases, we were able to identify candidate genes within these regions. Results from GWAS in humans and other species suggest that conformation traits are complex and affected by various factors such as bone and cartilage development, muscle growth, fat accumulation and body weight gain [17, 24, 25]. Changes in these factors and how they interact with each other probably determine the skeletal structure and movement pattern of an individual. Several of the identified candidate genes e.g. *LRPPRC*, *WRAP73*, *VRTN* and *PPARD* are involved in bone and muscle development at different levels. Liu and McKeehan [26] suggested that *LRPPRC* has a role in the regulation of cytoskeleton network activity by analyzing its sequence and the proteins it interacts with. *WRAP73* belongs to the WD repeat protein gene family, which includes the *WDR8* gene that plays an essential role in the ossification process. Expression of *WDR8* was observed in bone-forming cells and in bone and cartilage tissue during the early stage of ectopic ossification in mouse [27]. A polymorphism in the *VRTN* gene was found to be related to the number of vertebrae in domestic pigs with one allele resulting in an additional segment in the thoracic vertebrae compared with the wild type allele [28]. *PPARD,* a candidate gene for BACK and CONF in Landrace, was reported to be linked with muscle development and metabolism in pigs [29]. Expression of porcine *PPARD* inhibits the formation of myotube and increases adipocyte differentiation in mouse myoblasts [29]. In addition, several GWAS revealed the association of *PPARD* with limb bone length [30] and growth and fatness traits [31] in pigs. Similarly, *HMGA1* which is located in a QTL region that we identified on SSC7 in Duroc, was reported to be associated with length of limb bone in pigs [30, 32] and height and length of hip axis in humans [24]. In vitro studies showed that addition of *HMGA1* enhanced the proliferation of chondrocyte cells by regulating the expression of a chondrocyte-specific marker [33]. These findings suggest that *VRTN*, *PPARD* and *HMGA1* are candidate genes for conformation traits in pigs by regulating bone and muscle development.

Growth-promoting factors, including insulin and IGF, are known to participate in bone and fat metabolism and in the growth process [34, 35]. In this study, several genes involved in the growth pathway were identified, such as *IGF2BP2*, G*H1*, *CCND2* and *MSH2*. The protein IGF2BP2 plays an important role in controlling the action of IGF [36] which are associated with growth and fatness traits in pigs [31]. Similarly, GH1 is necessary for growth promotion and energy metabolism regulation [37]. Members of the candidate gene *CCND2* for BACK in Landrace are essential for growth of pancreatic islets [38], which play a role in the regulation of the growth of an animal via its hormones. Another gene related to the growth pathway that was identified in this study, *MSH2*, regulates the activity of the melanocortin system which is involved in fat accumulation, feed intake and daily weight gain in pigs [39]. These results suggest that growth-related genes have a regulatory function on conformation traits in pigs but how they are associated with each other needs further study.

In this study, we identified several SNPs and chromosomal regions that were significantly associated with the traits analyzed. However, some of the top SNPs were located in intergenic regions between coding genes. Some of these genes, such as *RIPPLY2* (SSC1: 92.6 Mb) and *SYNDIG1L* (SSC7: 103.5) are associated with vertebrae and rib development. *RIPPLY2* is involved in somitogenesis during the embryo stage in mice, and knockout mice for this gene die during the perinatal period due to severe vertebrae and rib malformation [40]. In humans, it was shown that mutations in *RIPPLY2* are associated with segment defects of the vertebrae [41]. In pigs, Verardo et al. [42] reported that *SYNDIG1L* was associated with number of teats while Duijvesteijn et al. [43] showed that number of teats and number of vertebras were controlled by several pleiotropic coding genes. These findings suggest that *SYNDIG1L* may have a role in the development of vertebrae in pigs. Many causal genetic factors have been previously reported to be located in the regulatory regions of genes which may explain why several candidate SNPs were identified at intergenic locations in this study. However, it may also indicate that the density of the SNP chips used was not sufficiently high. In that case, an association analysis with imputed whole-genome sequence might be able to further pinpoint the causal mutations [12].

The highly significantly associated gene with CONF in Duroc, *PRKCE*, is involved in several biological processes. PRKCE is a well-known key factor in cell proliferation and differentiation, muscle contraction, gene expression, cell growth and apoptosis, metabolism and diabetes, as reviewed by Akita [44] and Geraldes and King [45]. PRKCE plays an essential role in regulating lipogenesis by the interaction with GH [44] or IGF1 [46]. However, overexpression of *PRKCE* results in malignant tumors and diabetes [44, 46]. This relationship between growth-related factors and diabetes and bone metabolism could explain the association of *PRKCE* with conformation traits.

Not all candidate genes that were identified here have an obvious biological function on conformation traits. For example, some of the candidate genes are expressed in neuronal tissues and are related to the activity of the neuronal system, i.e. *SLC14A2*, *PPM1G* and *NGFR* [47–49]. The role of neuronal genes in the regulation of the metabolism, fat accumulation and body weight gain was investigated in humans and pigs [47, 50, 51]. Willer et al. [50] conducted a meta-analysis of 15 GWAS, which detected eight loci that are significantly associated with body mass index in humans. Another association study that combined human and pig data showed that several neuronal genes were associated with subcutaneous fat accumulation, which provides further support for a role of the nervous system on fat metabolism [47]. Another gene, *SLC14A2* which is a member of the SLC super family, is mainly expressed in brain [52] and associated with fat thickness in humans and pigs [47]. Since the fat accumulation process probably affects the body structure and movement pattern of an animal, it would be interesting to investigate further how the nervous system influences conformation traits.

### Within-breed multi-trait meta-analysis

In this study, the detection of QTL was enhanced in the multi-trait meta-analyses compared with the single-trait analyses. In other words, larger numbers of significant SNPs and higher significance levels of the top SNPs were observed in the meta-analyses. This phenomenon was also reported by Bolormaa et al. [22] for stature, fatness and reproduction related traits in beef cattle and more recently by Pausch et al. [53] for mammary gland morphology traits in dairy cattle. The ability to account for the relatedness between traits of multi-trait meta-analyses can explain the enhanced power of this approach here, where the four traits studied were highly genetically correlated [54]. The most significant SNPs detected in the multi-trait analyses for all three breeds reached higher significance level compared with the single-trait analysis, but they were not located within any annotated genes. Our results confirmed that multi-trait analyses can enhance the power to detect new associated SNPs, pleiotropic QTL and SNPs that were associated with only one of the correlated traits [55].

### Across-breed meta-analysis

Phenotype and genotype data from all three breeds were available, and thus a GWAS analysis with pooled data could have been done instead of the across-breed meta-analysis. However, these breeds have been separated for many generations and have undergone strong artificial selection and genetic drift, which means that different sets of QTL may be segregating in each breed. Moreover, GWAS relies on linkage disequilibrium (LD) between markers and causal variants. The marker-QTL linkage phase can differ by breed and, thus, a joint analysis will have less power to detect such QTL. The genome-wide and local pattern of LD and the persistence of LD phase have been investigated in these three breeds [56], which showed that the persistence of LD phase was higher between Landrace and Yorkshire than between these two breeds and Duroc.

The results of the meta-analysis confirmed the associations of a number of candidate genes that had been detected in the single-breed analyses, such as *PRKCE*, *SLC14A2*, *HMGA1*, *VRTN* and *SYNDIG1L*. The greater significance level of the top SNPs was expected due to the improved power of detection for common SNPs in the meta-analyses compared with single-breed analyses. This advantage of meta-analyses has been reported in pigs [32], cattle [57, 58], and human [24, 50]. In this study, meta-analyses revealed several new QTL regions and candidate genes associated with the traits studied among which some are linked with bone, skeletal or muscle development, including *SOS2*, *TRIM24* and *ELMO1*. Mutations in *SOS2* are associated with the Noonan syndrome in humans, i.e. patients with this syndrome have short stature, weak muscles and malformed skeleton [59]. They are often diagnosed with GH deficiency and respond well to GH therapy [60], which suggests that *SOS2* is associated with conformation traits via growth-regulated processes. *TRIM24* belongs to the superfamily of tripartite motif-containing proteins which have a role in the immune system. A member of this protein family, *TRIM76*, was reported to be highly expressed in porcine skeletal muscle and significantly associated with carcass traits such as ham percentage and intramuscular fat [61]. The results of our meta-analysis suggested that *ELMO1*, which is associated with the development and progress of diabetes nephropathy in humans, is a candidate gene for CONF [62]. Another interesting region associated with CONF was detected at 25 Mb on SSC12 where the Antp homeobox (HOX) gene family is located. Four *HOX* gene clusters (*HOXA*, *HOXB*, *HOXC* and *HOXD*) are known to be associated with the formation and development of vertebrae [63]. The combined expression of these genes defines somite identities in mammalian embryos, which direct the differentiation and the development of the vertebrae according to their location [63]. In this study, *HOXB5* and *HOXB13* were the closest genes to the top SNP in the associated region. An association between *HOXB* genes and number of lumbar and thoracolumbar vertebrae was also reported in pigs [64].

The diversity in the candidate genes and their biological functions found in this study confirmed the complex pattern of the genetic mechanisms that underlie conformation traits in pigs. Bone and skeleton development, muscle and fat metabolism and growth processes probably interact together to determine the general conformation and movement of a pig. However, these interactions are still unclear and should be investigated further.

## Conclusions

Conformation traits are complex and appear to be controlled by genes that are involved in different biological processes, including bone and skeleton development, muscle and fat metabolism and body growth. Our results suggested the association of the *LRPPRC*, *WRAP73*, *VRTN*, *PPARD*, *IGF2BP2*, *GH1*, *CCND2* and *MSH2* genes with conformation traits in pigs. We show that meta-analysis is a powerful QTL detection approach since we were able to detect possible QTL with pleiotropic effects in the multi-trait meta-analyses, and novel relevant candidate genes such as *SOS2*, *TRIM24* and *ELMO1* in the across-breed meta-analyses. Our findings are reliable and can be used in fine-mapping to confirm the effects of the genes identified, as well as in marker-assisted selection to improve the conformation in pigs.

## Additional files

**Additional file 1: Figure S1.** Manhattan plot of GWAS in Landrace pigs for (a) FRONT, (b) BACK, (c) HIND and (d) CONF. The data provided represent the Manhattan plot of single-trait association analyses in Landrace pigs for four traits studied.

**Additional file 2: Figure S2.** Manhattan plot of GWAS in Yorkshire pigs for (a) FRONT, (b) BACK, (c) HIND and (d) CONF. The data provided represent the Manhattan plot of single-trait association analyses in Yorkshire pigs for four traits studied.

**Additional file 3: Figure S3.** Manhattan plot of GWAS in Duroc pigs for (a) FRONT, (b) BACK, (c) HIND and (d) CONF. The data provided represent the Manhattan plot of single-trait association analyses in Duroc pigs for four traits studied.

**Additional file 4: Figure S4.** Manhattan plot of within-breed multi-trait meta-analyses in (a) Landrace, (b) Yorkshire and (c) Duroc. The data provided represent the Manhattan plot of within-breed multi-trait meta-analyses in three breeds studied.

**Additional file 5: Table S1.** QTL regions and the most significant SNP within each region in across-breed meta-analyses. The data provided represent the QTL regions and the information of the most significant SNP within each region in across-breed meta-analyses.
