# Supplementary material for: Genome-wide association study for conformation traits in three Danish pig breeds
Source: Genet Sel Evol. 2017 Jan 24;49:12. doi: 10.1186/s12711-017-0289-2 (PMC5259967; doi:10.1186/s12711-017-0289-2)
Supplement: Supplementary file 1 — Additional file 1: Figure S1. Manhattan plot of GWAS in Landrace pigs for (a) FRONT, (b) BACK, (c) HIND and (d) CONF. The data provided represent the Manhattan plot of single-trait association analyses in Landrace pigs for four traits studied. [file 12711_2017_289_MOESM1_ESM.docx]

| **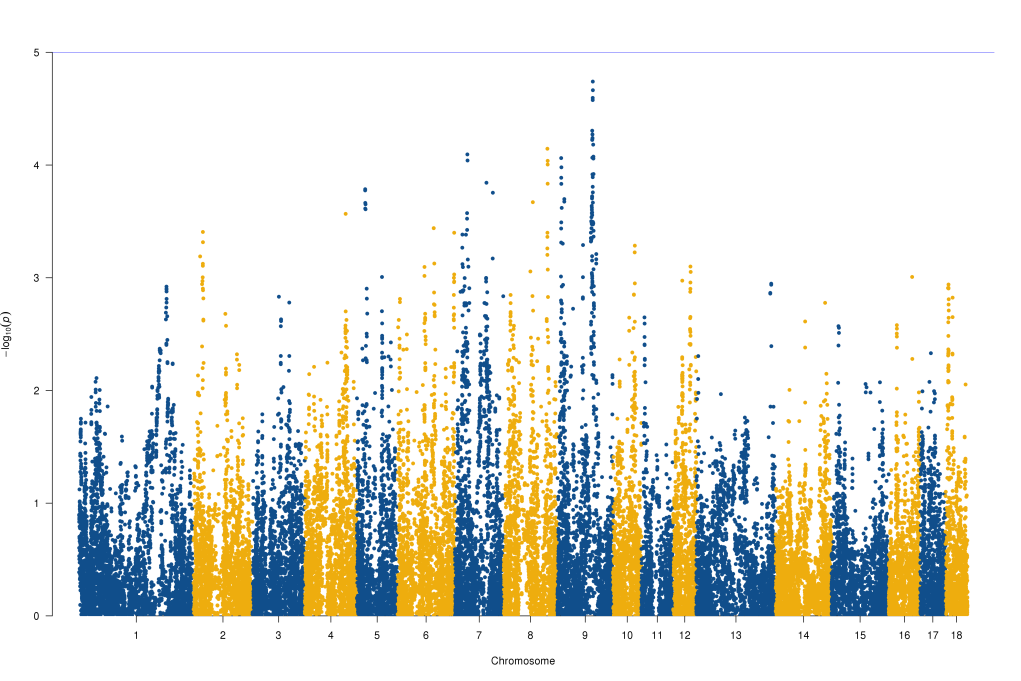**  **a** (λ=1.23) | **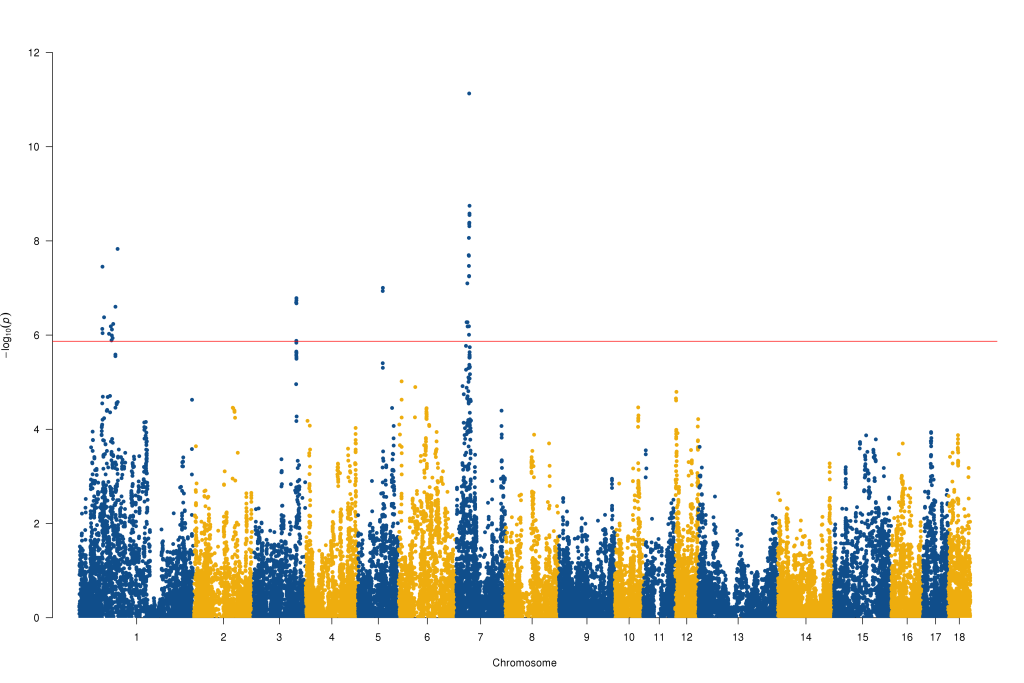**  **b** (λ=1.60) |
| --- | --- |
| **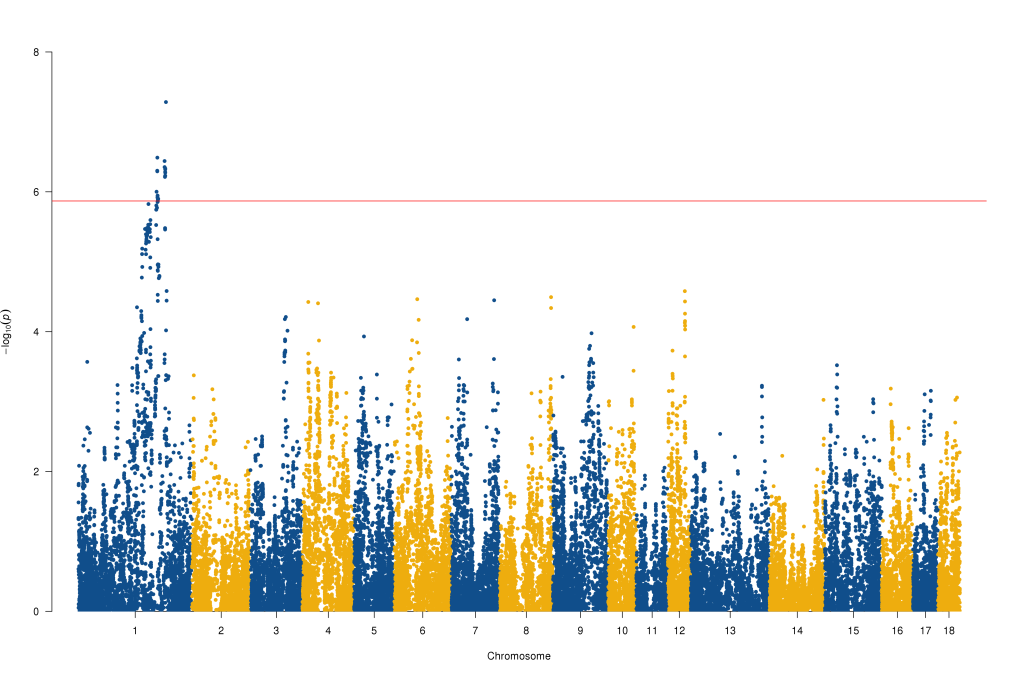**  **c** (λ=1.46) | **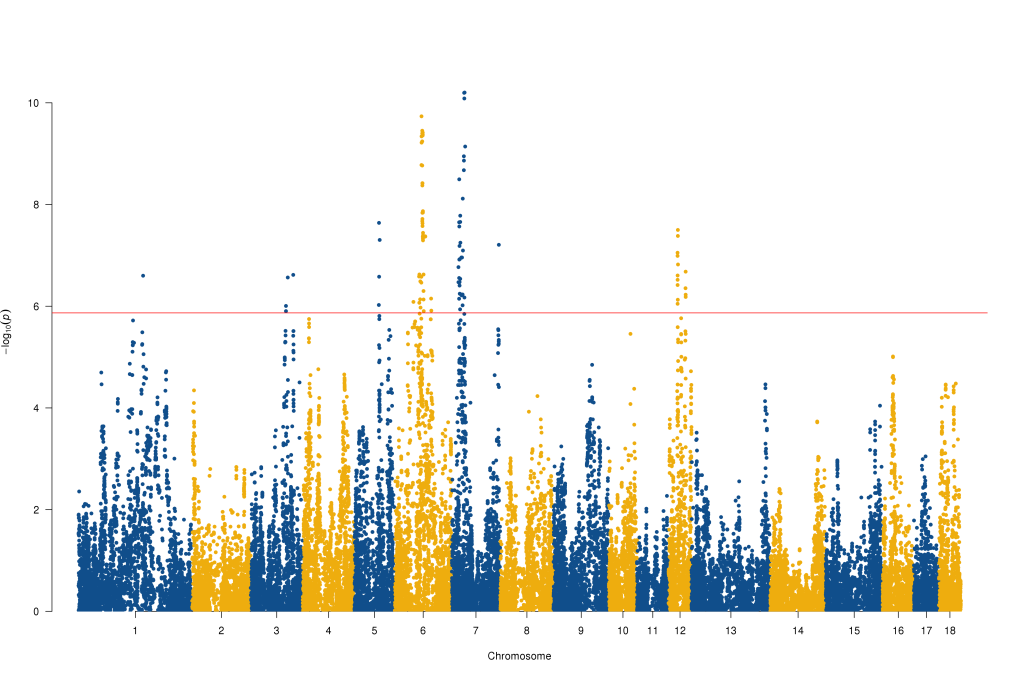**  **d** (λ=1.96) |

**Fig. S1 Manhattan plot of GWAS in Landrace pigs for (a) FRONT, (b) BACK, (c) HIND and (d) CONF**

x-axis represents chomosomes and y-axis represents ${-log}_{10}(P-value)$. The blue and red line indicate genome-wide significance threshold at P-value <5x10^-5^ and P-value <1.38x10^-6^ , respectively. The genomic inflation factor (λ) was presented in the brackets.
