# Supplementary material for: Genome-wide association study for conformation traits in three Danish pig breeds
Source: Genet Sel Evol. 2017 Jan 24;49:12. doi: 10.1186/s12711-017-0289-2 (PMC5259967; doi:10.1186/s12711-017-0289-2)
Supplement: Supplementary file 4 — Additional file 4: Figure S4. Manhattan plot of within-breed multi-trait meta-analyses in (a) Landrace, (b) Yorkshire and (c) Duroc. The data provided represent the Manhattan plot of within-breed multi-trait meta-analyses in three breeds studied. [file 12711_2017_289_MOESM4_ESM.docx]

| **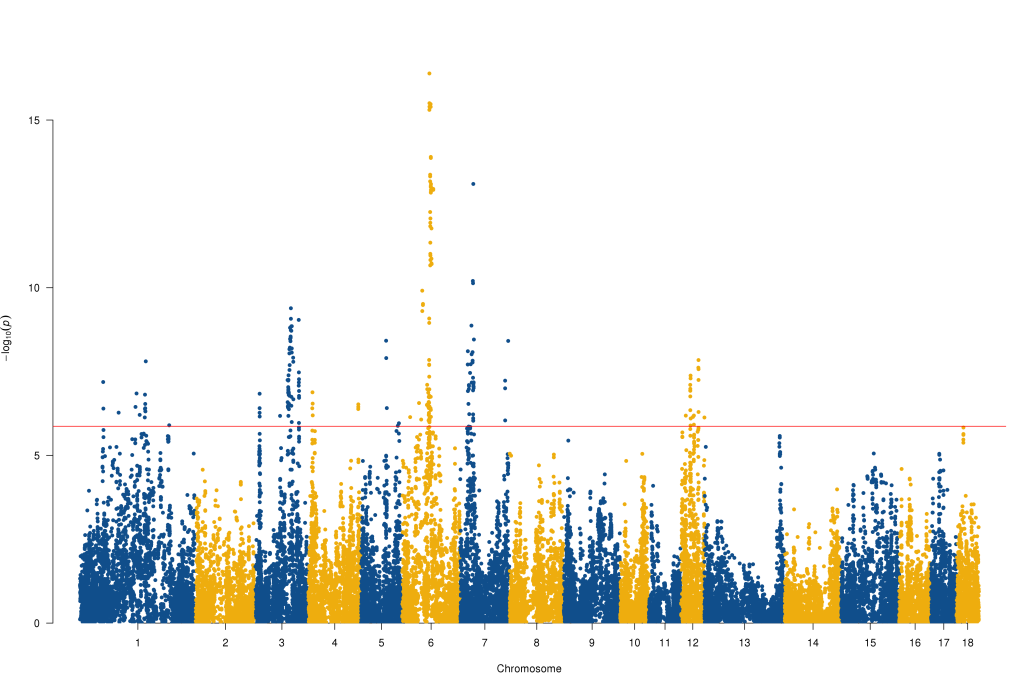**  **a** | **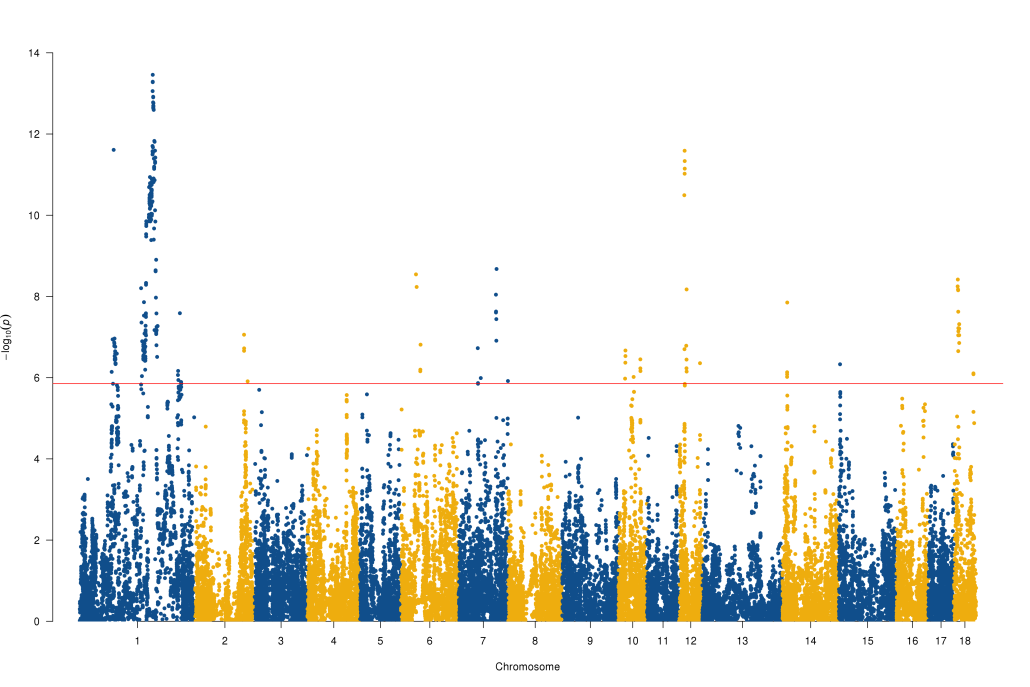**  **b** |
| --- | --- |
| **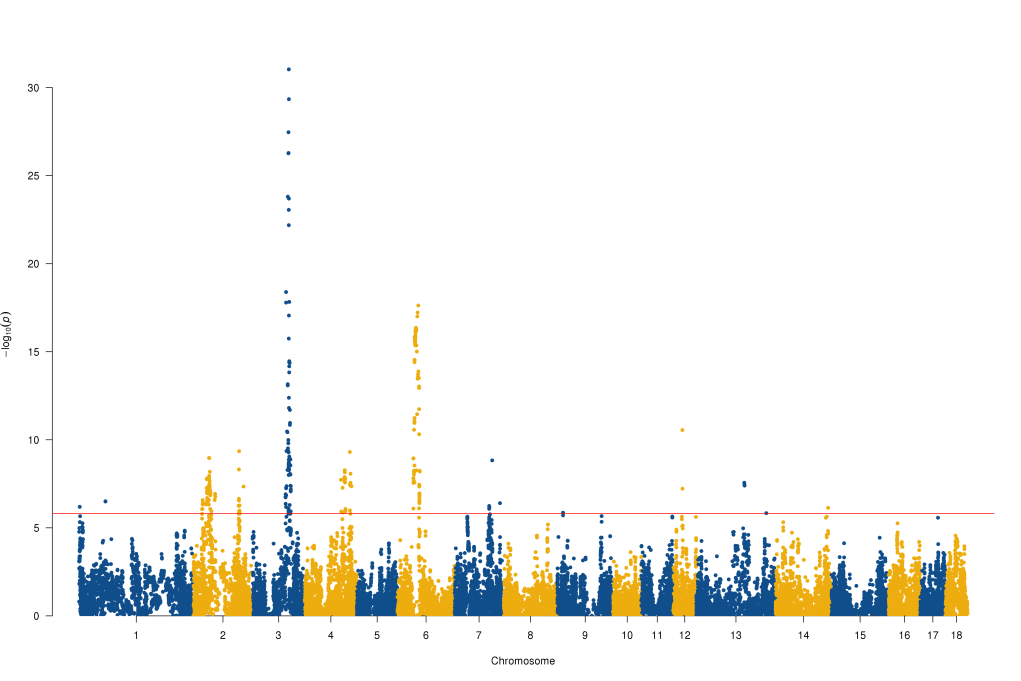**  **c** |  |

**Fig. S4 Manhattan plot of within-breed multi-trait meta-analysis in (a) Landrace, (b) Yorkshire and (c) Duroc**

x-axis represents chomosomes and y-axis represents ${-log}_{10}(P-value)$. The red line indicates genome-wide significance threshold at P-value <1.38x10^-6^.
