# Supplementary material for: Genome-wide association study for conformation traits in three Danish pig breeds
Source: Genet Sel Evol. 2017 Jan 24;49:12. doi: 10.1186/s12711-017-0289-2 (PMC5259967; doi:10.1186/s12711-017-0289-2)
Supplement: Supplementary file 5 — Additional file 5: Table S1. QTL regions and the most significant SNP within each region in across-breed meta-analyses. The data provided represent the QTL regions and the information of the most significant SNP within each region in across-breed meta-analyses. [file 12711_2017_289_MOESM5_ESM.docx]

**Table S1. QTL regions and the most significant SNP within each region in across-breed meta-analysis**

| Trait | n | Chr | Region^a^ | Right position, bp | Left position, bp | Most significant SNP | | | Candidate genes^c^ |
| --- | --- | --- | --- | --- | --- | --- | --- | --- | --- |
|  |  |  |  |  |  | SNP^b^ | Position, bp | P-value |  |
| FRONT | 8 | 3 | 1 | 100448894 | 100448894 | rs81373756 | 100448894 | 6.61x10^-7^ | *PRKCE* |
|  |  | 6 | 1 | 51775907 | 57685509 | rs81226413 | 55763196 | 4.22x10^-8^ | *ENSSCT00000003685* |
|  |  | 7 | 1 | 34978383 | 34978383 | rs342640079 | 34978383 | 5.80x10^-8^ | GRM4, HMGA1 |
| BACK | 56 | 1 | 1 | 63850852 | 63850852 | rs80850790 | 63850852 | 8.52x10^-8^ | SRSF12, PNRC1 |
|  |  | 1 | 2 | 105362894 | 105362894 | rs81287678 | 105362894 | 1.48x10^-8^ | *SLC14A2* |
|  |  | 1 | 3 | 147989923 | 147989923 | rs81348555 | 147989923 | 7.37x10^-7^ | RASGRP1 |
|  |  | 3 | 1 | 99466921 | 100448894 | rs81327799 | 99466921 | 8.65x10^-13^ | EPCAM, C2orf61 |
|  |  | 3 | 2 | 118746225 | 118746225 | rs81234106 | 118746225 | 8.16x10^-9^ | *NRBP1* |
|  |  | 6 | 1 | 45906794 | 45906794 | rs81274518 | 45906794 | 4.70x10^-13^ | *ENSSSCT00000003376* |
|  |  | 7 | 1 | 27576016 | 36202231 | rs342640079 | 34978383 | 6.55x10^-9^ | GRM4, HMGA1 |
|  |  | 18 | 1 | 11430257 | 12218768 | rs81256422 | 11430257 | 5.10x10^-7^ | *ATP6V0A4* |
| HIND | 69 | 1 | 1 | 103940473 | 103980217 | rs80904741 | 103980217 | 6.11x10^-7^ | ENSSSCG00000025743 |
|  |  | 1 | 2 | 199041898 | 200722738 | rs318336483 | 200350940 | 1.22x10^-10^ | *SOS2* |
|  |  | 1 | 3 | 216980027 | 224959989 | rs80908482 | 218883528 | 1.13x10^-8^ | CAAP1 |
|  |  | 1 | 4 | 239238226 | 242984908 | rs80789031 | 239238226 | 4.67x10^-12^ | PTPRD |
|  |  | 6 | 1 | 43110092 | 59780811 | rs81333163 | 57685509 | 1.45x10^-9^ | A1BG, RPS5 |
|  |  | 7 | 1 | 103495170 | 121907514 | rs80894106 | 103495170 | 1.24x10^-9^ | VRTN, SYNDIG1L |
|  |  | 18 | 1 | 11617196 | 11928951 | rs81470637 | 11617196 | 2.56x10^-7^ | *TRIM24, ENSSCG00000021753* |
| CONF | 235 | 1 | 1 | 94490355 | 103980217 | rs80904741 | 103980217 | 3.08x10^-8^ | ENSSSCG00000025743 |
|  |  | 1 | 2 | 176949944 | 212600506 | rs80985915 | 179235127 | 1.23x10^-12^ | *CPLX4* |
|  |  | 1 | 3 | 239139595 | 243293703 | 36938 | 243293703 | 1.20x10^-9^ | SLC1A1, ENSSSCG00000030006 |
|  |  | 2 | 1 | 2673647 | 2673647 | rs81357266 | 2673647 | 7.11x10^-8^ | CCND1, TPCN2 |
|  |  | 2 | 2 | 42398783 | 42816595 | rs81329722 | 42398783 | 1.05x10^-6^ | ENSSSCG00000026074, ENSSSCG00000029992 |
|  |  | 3 | 1 | 100448894 | 101373710 | rs81373942 | 101373710 | 1.07x10^-13^ | SRBD1, SIX2 |
|  |  | 4 | 1 | 107356545 | 109885605 | 58957 | 109319576 | 3.03x10^-8^ | PDZK1, ENSSCG00000006692 |
|  |  | 6 | 1 | 50079246 | 57685509 | rs81344309 | 52085979 | 1.34x10^-20^ | ENSSCG00000003243, ZNF614 |
|  |  | 6 | 2 | 68263560 | 74657757 | rs81389001 | 74354607 | 1.20x10^-9^ | ENSSSCG00000003521, ENSSSCG00000003522 |
|  |  | 7 | 1 | 34978383 | 34978383 | rs342640079 | 34978383 | 3.88x10^-17^ | GRM4, HMGA1 |
|  |  | 7 | 2 | 103495170 | 103495170 | rs80894106 | 103495170 | 1.68x10^-16^ | VRTN, SYNDIG1L |
|  |  | 7 | 3 | 130889396 | 131126819 | rs81397155 | 131126819 | 5.03x10^-10^ | TDRD9, CEP170B |
|  |  | 12 | 1 | 24901250 | 25708148 | rs81270042 | 24901250 | 7.68x10^-9^ | HOXB5,HOXB13 |
|  |  | 12 | 2 | 62421422 | 63239985 | rs81222510 | 63095062 | 1.73x10^-10^ | NT5M, MED9 |
|  |  | 13 | 1 | 99515364 | 99515364 | rs81255191 | 99515364 | 6.53x10^-7^ | P2RY14, P2RY12 |
|  |  | 13 | 2 | 135513903 | 135513903 | rs81478619 | 135513903 | 5.05x10^-7^ | ENSSSCG00000011811 |
|  |  | 18 | 1 | 9044109 | 12638310 | rs81319580 | 12218768 | 2.40x10^-9^ | *CREB3L2* |
|  |  | 18 | 2 | 40830282 | 40882202 | rs81468980 | 40855282 | 8.88x10^-8^ | *ELMO1* |

*FRONT* Front leg quality, *BACK* Back quality, *HIND* Hind leg quality, *CONF* Overall conformation**;** *n* Number of significant SNPs; *Chr Sus scrofa* chromosome

^a^ *Region* QTL region within a chromosome

^b^ *SNP* rsID

^c^ *Candidate genes* in which the most significant SNPs located are in italic
